# How Silks Are Given Weight

**Published:** 1882-03

**Authors:** 


### HOW SILKS ARE GIVEN WEIGHT.

IN the report of Sir Edward Thornton, lately Minister at Washington, and now Ambassador at St. Petersburg, attention is drawn to certain mysterious fires, both in warehouses and aboard ship, which, after careful inquiry by a police committee and a board of underwriters in New York, have been traced to consignments of black silk. The immediate cause of danger is, it appears, the chemical materials now used to give weight as well as improved color to the silks. The art, says the report, has reached such perfection that the weight of the natural silk can be increased four-fold without apparent adulteration ; but the minerals, vegetables, acids and alkalies thus used, combined with animal substances and the natural germ of the silk, constitute a fermentable compound which generates carbonization or combustion under pressure, confinement and heat. That the black silk goods have ignited spontaneously from these causes and caused serious fires is considered to be abundantly proved by the evidence.—*London News.*

### TWO SIDES OF A STORY.

Two boys went to hunt grapes. One was happy because they found grapes. The other was unhappy because the grapes had seeds in them.

Two men, being convalescent, were asked how they were. One said, "I am better to-day." The other said, "I was worse yesterday."

When it rains one man says, "This will make mud." Another, "This will lay the dust."

Two children looking through colored glasses, one said, "The world is blue." The other said, "It is bright."

A servant thinks a man's house is principally kitchen. A guest, that it is principally parlor.

"I am sorry that I live," says one man. "I am sorry that I must die," says another.

"I am glad," says one, "that it is no worse." "I am sorry," says another, "that it is no better."

One man counts everything that he has a gain. Another counts everything else than he receives a loss.

Two boys eating their dinner, one said, "I would rather have something or other than this." The other said, "This is better than nothing.

One man spoils a good repast by thinking of a better repast of another. Another one enjoys a poor repast by contrasting it with none at all.

One man is thankful for his blessings. Another is morose for his misfortunes.

One man thinks he is entitled to a better world, and is dissatisfied because he hasn't got it. Another thinks he is not justly entitled to any, and is satisfied with this.

One man makes up his accounts from his wants. Another from his assets.

---

WE educate our children, as the saying goes, but they also do much for us in the same way. They open our eyes and "hold a mirror up to nature;" indeed, I have sometimes thought that children have the "giftie gie us," and we see ourselves at least as they see us. A little boy said to his mother, the night before Christmas, "Mamma, you and my father are invited to my kindergarten Christmas-tree; you are to come at 10 o'clock, but I"—this with a delightful air of importance—"I must go as early as 6, to fuss around like women." The mother to whom this was said thought with humiliation that the artlessly spoken sentence actually described her manner of doing things, and it induced a great many thoughts for which she hopes she is better. How much that we do has the appearance and really is worthy of no better title than "fussing around!" How much needless anxiety we give ourselves! There are few women who can move calmly forward about their work, doing the best they can, without fretting and bustling about in a way that disturbs the peace of the household. The average woman—as people are fond of calling all but the exceptionally great—might imitate the average man in this respect with great gain; for there are comparatively few men who do not learn at an early age that they cannot afford to be disturbed by every little unexpected disagreeable occurrence. We see clearly that it is an evidence of weakness in a man, and isn't it just the same thing in us? E. W. B.

---

A PHRENOLOGIST was the victim of a mean trick by a Washington man. The latter had three photographs of himself taken, in one of which he appeared dressed as a woman; in another as a well-to-do architect; in the third as a spectacled professor. Then he sent the photographs on for phrenological description. He received in a few days three charts. The "architect" was described as an impulsive person, of strong lung power, etc.; the "woman" seemed to have less lung power than the architect, and appeared "able to convert nutritious materials into healthy nutriment rapidly and abundantly." The two were well adapted to marriage. As to the "professor," he was described as unfit to marry, being of uncongenial temperament, etc. The joke is considered a large one on phrenology.

